# Whole blood transcriptional profiling comparison between different milk yield of Chinese Holstein cows using RNA-seq data

**DOI:** 10.1186/s12864-016-2901-1

**Published:** 2016-08-22

**Authors:** Xue Bai, Zhuqing Zheng, Bin Liu, Xiaoyang Ji, Yongsheng Bai, Wenguang Zhang

**Affiliations:** 1 College of Animal Science, Inner Mongolia Agricultural University, Hohhot, 010018 China; 2 Institute of ATCG Nei Mongol Bio-Information, Hohhot, 010020 China; 3 Nei Mongol BioNew Technology Co.Ltd, Hohhot, 010018 China; 4 Department of Biology, Indiana State University, Terre Haute, IN 47809 U.S.A.; 5 Kunming Institute of Zoology, Chinese Academy of Sciences, Kunming, China

**Keywords:** Whole blood, Immune response, Milk yield, Differentially expressed genes, Dairy cattle

## Abstract

**Background:**

The objective of this research was to investigate the variation of gene expression in the blood transcriptome profile of Chinese Holstein cows associated to the milk yield traits.

**Results:**

We used RNA-seq to generate the bovine transcriptome from the blood of 23 lactating Chinese Holstein cows with extremely high and low milk yield. A total of 100 differentially expressed genes (DEGs) (*p* < 0.05, FDR < 0.05) were revealed between the high and low groups. Gene ontology (GO) analysis demonstrated that the 100 DEGs were enriched in specific biological processes with regard to defense response, immune response, inflammatory response, icosanoid metabolic process, and fatty acid metabolic process (*p* < 0.05). The KEGG pathway analysis with 100 DEGs revealed that the most statistically-significant metabolic pathway was related with Toll-like receptor signaling pathway (*p* < 0.05). The expression level of four selected DEGs was analyzed by qRT-PCR, and the results indicated that the expression patterns were consistent with the deep sequencing results by RNA-Seq. Furthermore, alternative splicing analysis of 100 DEGs demonstrated that there were different splicing pattern between high and low yielders. The alternative 3’ splicing site was the major splicing pattern detected in high yielders. However, in low yielders the major type was exon skipping.

**Conclusion:**

This study provides a non-invasive method to identify the DEGs in cattle blood using RNA-seq for milk yield. The revealed 100 DEGs between Holstein cows with extremely high and low milk yield, and immunological pathway are likely involved in milk yield trait. Finally, this study allowed us to explore associations between immune traits and production traits related to milk production.

**Electronic supplementary material:**

The online version of this article (doi:10.1186/s12864-016-2901-1) contains supplementary material, which is available to authorized users.

## Background

Milk yield and milk composition of lactating cows are the most important economic traits in dairy cattle. In the past century, genetic selection on milk yield has improved milk production in cattle. With the development of quantitative trait loci (QTLs), genome-wide association studies (GWAS) and RNA sequencing (RNA-seq) technologies, a large number of candidate genes and SNPs associated with milk performance traits have been identified, such as *DGAT1* and *GHR* genes [1–6]. Previous studies described the associations between *DGAT1* K232A polymorphism and milk production traits [7, 8]. Blott et al. identified a significant SNP (BFGL-NGS-118998) inside the *GHR* gene that has an important role related to milk traits [9].

Mammary gland is an important organ to synthesize and secrete milk. The mammary epithelial cell has a remarkable ability to convert blood circulating nutrients into milk components [10]. Thus, almost all the studies related to milk performance traits are based on mammary gland. For example, Cui et al. collected mammary gland samples from four lactating cows after slaughter. They used RNA-seq to generate the bovine mammary transcriptome with high and low milk fat and protein percentage [11]. Finucane et al. compared bovine mammary expression profiles before and after parturition using microarray [12]. However, there are still limitations in analysis of milk performance traits using bovine mammary tissue, such as sampling difficultly, high cost of sampling and tremendous damage to the lactating cows, which resulted in small sample size.

Milk is composed of fat, protein, lactose, minerals, vitamins and water, and all these nutrients derive from blood [10, 13–15]. Some data shows that synthesizing 1L milk requires 400-500L blood flow though the mammary gland. So plenty of blood is essential for synthesizing milk. Previous studies associated with milk traits were only focused on blood physiological and biochemical indexes, and there are very few studies associating gene expression with milk traits in cattle blood. A gene expression profile from blood provides new opportunities to clarify the basic mechanisms of complex traits in cattle milk. Besides, whole blood is a complex mixture of cells and can accurately reflect the physiological condition and health level of cows. Many studies have used blood to diagnose disease in dairy cattle, such as mastitis [16, 17]. Most importantly, blood is easier to sample in comparison with other tissues and involves limited handling of animals. Furthermore, the lactation process requires multiple tissues and organs to complete, such as mammary gland, liver and adipose tissue. Hence, blood has the potential ability to represent milk performance traits more directly and comprehensively. Sandri et al. (2015) analyzed the gene expression profile in the blood related to the gene merit for milk productive traits using microarray [18]. Marcel et al. analyzed porcine peripheral blood mononuclear cells transcription profile of pigs with divergent humoral immune response and lean growth performance [19]. To complete understand the impact of blood transcriptome profiles on milk yield, comprehensive cataloguing of gene expression change within high yielders and low yielders is required. The aim of this study is to compare gene expression profiles in bovine whole blood of high and low milk yield cows, and to investigate potential molecular biomarkers in the blood transcriptome that relate to the productive potential of lactating cows using RNA-seq techniques.

## Results

### Analysis of expressed transcripts between high and low yielders

From 23 samples, we obtained total 74.6 Gb RNA-Seq data files (26,763,546 to 51,313,614 paired-end reads per sample). Nearly 68 % of the reads were mapped to the bovine genome UMD3.1.66 and approximately 62 % of the reads in every individual were uniquely mapped to the bovine genome. The alignment information for each sample is presented in Table 1. Of these, 16,314 and 16,151 expressed transcripts were revealed in high yielders and low yielders, respectively (Additional file 1: Table S1).

**Table 1:** Summary of the mapping information for each sample

Sample name	Total reads	Total mapped	Multiple mapped	Uniquely mapped
L1	51313614	37633474 (73.34 %)	1891840 (3.69 %)	35741634 (69.65 %)
L2	38408586	28132542 (73.25 %)	1626290 (4.24 %)	26506252 69.01 %)
L3	38156116	23779230 (62.32 %)	1254082 (3.29 %)	22525148 (59.03 %)
L4	28642682	18996768 (66.32 %)	873594 (3.05 %)	18123174 (63.27 %)
L5	29417132	18996768 (64.58 %)	2319892 (7.89 %)	16676876 (56.69 %)
L6	34296982	24799284 (72.31 %)	1048878 3.06%)	23750406 (69.25 %)
L7	28755318	16604970 (57.75 %)	896548 (3.12 %)	15708422 (54.63 %)
L8	31712370	21846718 (68.89 %)	980318 (3.09 %)	20866400 (65.80 %)
L9	28387782	17806510 (62.73 %)	917208 (3.24 %)	16889302 (59.49 %)
L10	32217694	20532020 (63.73 %)	896152 (2.78 %)	19635868 (60.95 %)
H1	27038432	19212408 (71.06 %)	944260 (3.50 %)	18268148 (67.56 %)
H2	32458530	23913728 (73.67 %)	999786 (3.08 %)	22913942 (70.59 %)
H3	31355676	23459842 (74.82 %)	933980 (2.98 %)	22525862 (71.84 %)
H4	32236792	22698066 (70.41 %)	949798 (2.95 %)	21748268 (67.46 %)
H5	31144146	20791688 (66.76 %)	1004534 (3.23 %)	19787154 63.53 %)
H6	32072196	22910946 (71.44 %)	1293062 (4.04 %)	21617884 (67.40 %)
H7	38188984	25973682 (68.01 %)	1154850 (3.02 %)	24818832 64.99 %)
H8	32100360	23224306 (72.35 %)	945914 (2.95 %)	22278392 (69.40 %)
H9	30227962	20288464 (67.12 %)	997090 (3.30 %)	19291374 (63.82 %)
H10	29321524	19085218 (65.09 %)	1073550 (3.66 %)	18011668 (61.43 %)
H11	32631714	21820588 (66.87 %)	1021428 (3.13 %)	20799160 (63.74 %)
H12	26763546	14871754 (55.57 %)	748546 (2.80 %)	14123208 (52.77 %)
H13	28755192	16809674 (58.46 %)	870238 (3.03 %)	15939436 (55.43 %)

### DEGs and splice events between high and low yielders

**Fig. 1:**
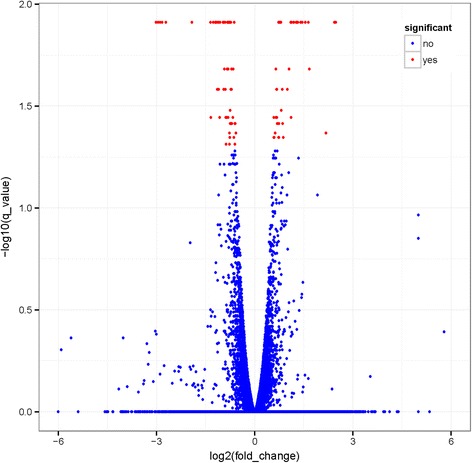
Volcano plot displaying DEGs between the whole blood of 10 low yielders and 13 high yielders. The y-axis corresponds to the mean expression value of log10 (q-value), and the x-axis displays the log2 fold change value. The red dots circled in the frame represent the significantly differentially expressed transcripts (p value < 0.05 and FDR < 0.05) between high and low milk yield cows; the blue dots represent the transcripts whose expression levels did not reach statistical significance between the two groups

**Fig. 2:**
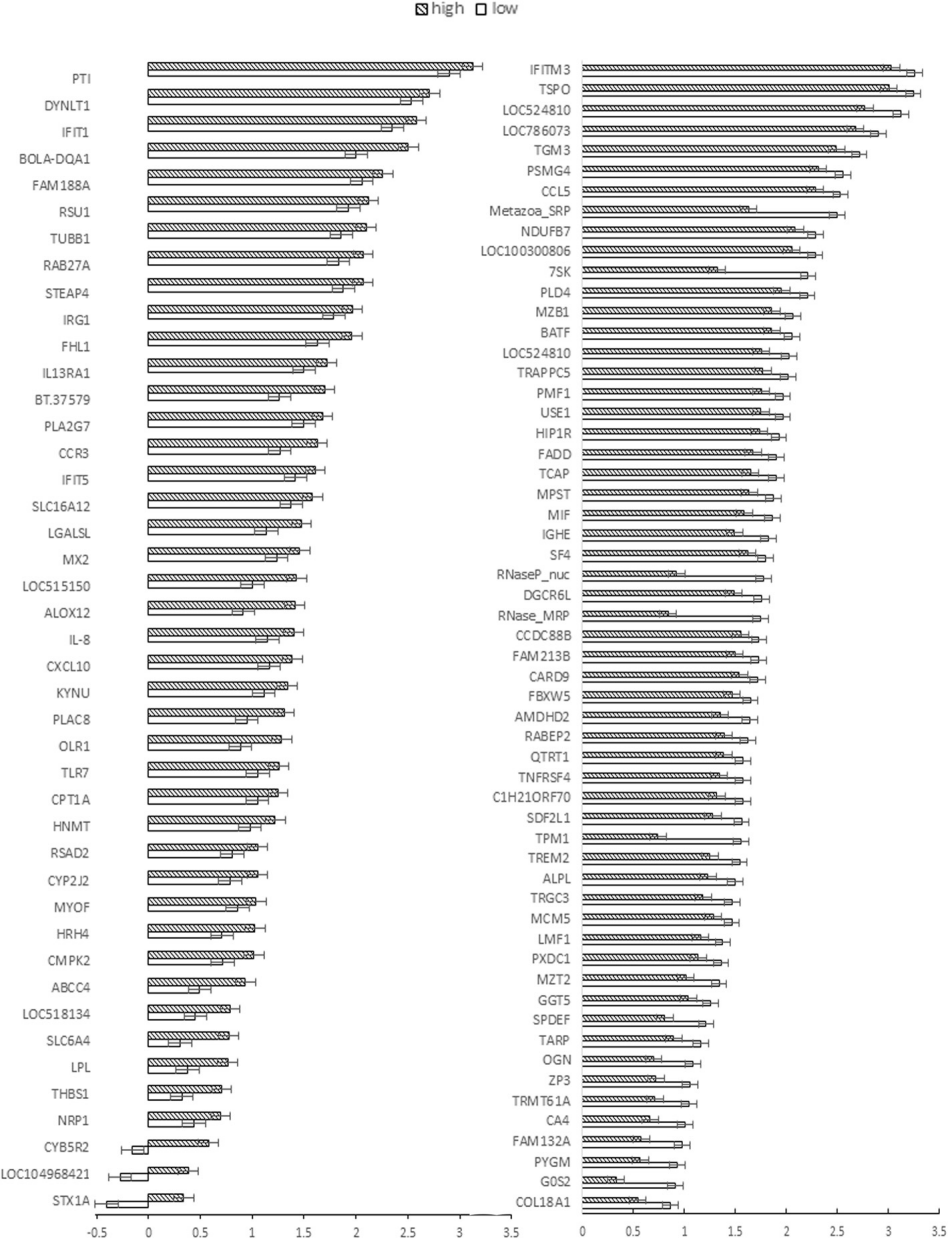
Expression of the 100 DEGs in bovine blood. The x-axis shows the gene expression level value of log10 (FPKM); y-axis shows the gene names. The left shows the 43 genes expressed higher in high yielders, and the right shows the rest 57 genes expressed higher in low yielders

To provide a better understanding of the biological mechanism of milk yield, it is essential to identify the DEGs between high and low milk yield cows. Based on the Cuffdiff analysis, a total of 100 DEGs (*p*<0.05, FDR< 0.05) were examined between the high and low yielders (Fig. 1). All the DEGs were located in chromosomes randomly, but there were no DEGs identified in chromosome 14, 20, 27, and 28. The expression level of 100 DEGs was from 2 to 1063 FPKM in high group, and 0.4 to 1794 FPKM in low group. In addition, 43 of the 100 DEGs were highly expressed in the high yielders; whereas, the other 57 DEGs showed lower expression in low yielders. The expression level of 100 DEGs is shown in Fig. 2 and the detail information is presented in Additional file 2: Table S2.

**Fig. 3:**
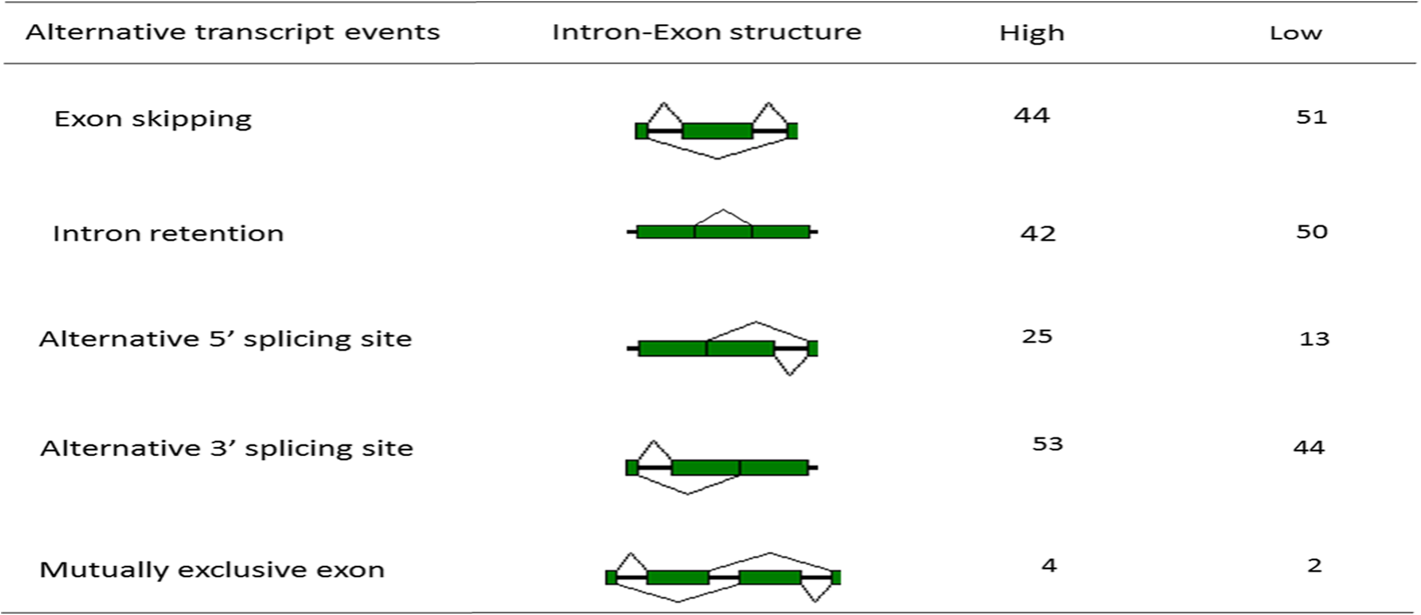
Statistics of mainly alternative splicing events. The first column shows the patterns of alternative splicing events; the second column shows the intron-exon structure, third and fourth column shows the number of AS events in high and low milk yield cows, respectively

Splice events are thought to contribute to phenotypic complexity during the mammalian evolution [20]. In total, we obtained 44,572 and 36,467 splice events in high and low yielders, respectively, compared to the annotated bovine genome UMD3.1.66. Of these, 214 (in high yielders) and 202 (in low yielders) differentially expressed splice events were identified. Further analysis showed that the 214 and 202 splice events involved 59 DEGs and 57 DEGs, respectively. Major splicing events such as, exon skipping (ES), intron retention (IR), alternative 5’ splicing site (A5SS), alternative 3’ splicing site (A3SS) and mutually exclusive exon (MXE) were detected in our studied bovine blood transcriptomes. The A3SS was the major splicing pattern observed in high group; but in low group, the major type was ES (Fig. 3). This suggests that high milk yield cows are more inclined to take the A3SS pattern. In addition, more splice sites were found in chromosome 26 in both groups.

### Functional classification of DEGs

**Fig. 4:**
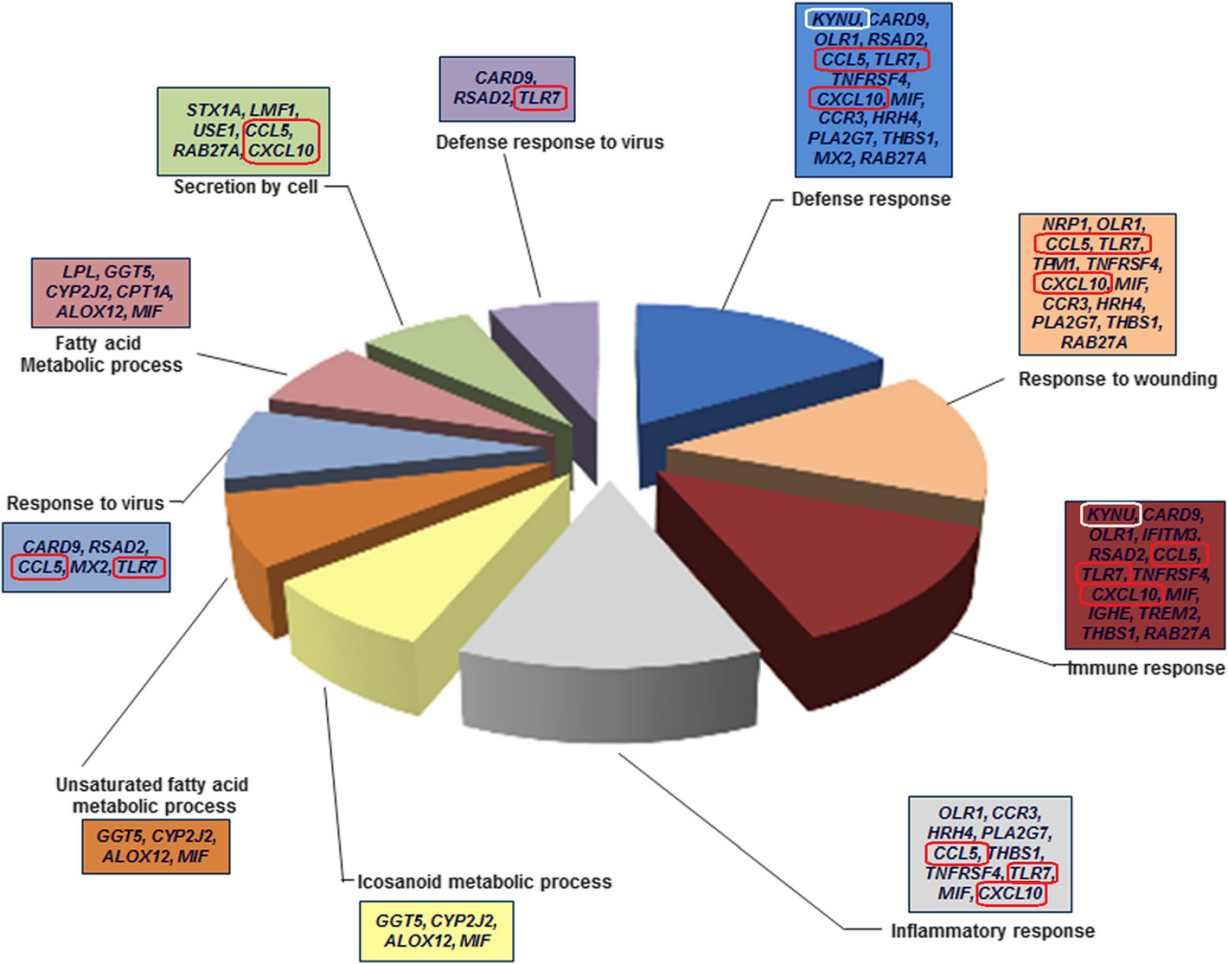
Diagram showed DEGs from the top 10 GO functional annotations in blood samples. Genes circled in red were also enriched in KEGG pathway analysis

The DAVID tool [21] was used to annotate the function of the 100 DEGs with the particular categories focusing on the gene ontology (GO) and Kyoto Encyclopedia of Genes and Genomes (KEGG) pathway. A total of 55 clusters (*p*<0.05) were significantly annotated with GO terms within three major function groups: biological process (BP), cellular component (CC), and molecular function (MF). The most significant GO categories observed were defense response, immune response, inflammatory response, icosanoid metabolic process, and fatty acid metabolic process (*p*<0.05) (Fig. 4). Only one KEGG pathway was enriched, which was the Toll-like receptor signaling pathway. On the other hand, based on the enrichment analysis of DEGs containing alternative splice sites revealed that the PPAR signaling pathway was only detected in the high yielded group. Detailed information of the DEG functional annotations are showed in Table 2.

**Table 2:** Gene Ontology and KEGG pathway annotation of DEGs between two groups

Category	GO ID	GO term	P value	No.of DEGs	DEGs
BP	GO:0006952	defense response	0.000001	15	KYNU, CARD9, OLR1, RSAD2, CCL5, TLR7, TNFRSF4, CXCL10, MIF, CCR3, HRH4, PLA2G7, THBS1, MX2, RAB27A
BP	GO:0009611	response to wounding	0.000008	13	NRP1, OLR1, CCL5, TLR7, TPM1, TNFRSF4, CXCL10, MIF, CCR3, HRH4, PLA2G7, THBS1, RAB27A
BP	GO:0006955	immune response	0.000024	14	KYNU, CARD9, OLR1, IFITM3, RSAD2, CCL5, TLR7, TNFRSF4, CXCL10, MIF, IGHE, TREM2, THBS1, RAB27A
BP	GO:0006954	inflammatory response	0.000027	10	OLR1, CCR3, HRH4, PLA2G7, CCL5, THBS1, TNFRSF4, TLR7, MIF, CXCL10
BP	GO:0006690	icosanoid metabolic process	0.001543	4	GGT5, CYP2J2, ALOX12, MIF
BP	GO:0033559	unsaturated fatty acid metabolic process	0.001954	4	GGT5, CYP2J2, ALOX12, MIF
BP	GO:0009615	response to virus	0.001958	5	CARD9, RSAD2, CCL5, MX2, TLR7
BP	GO:0006631	fatty acid metabolic process	0.002770	6	LPL, GGT5, CYP2J2, CPT1A, ALOX12, MIF
BP	GO:0032940	secretion by cell	0.003353	6	STX1A, LMF1, USE1, CCL5, RAB27A, CXCL10
BP	GO:0051607	defense response to virus	0.004209	3	CARD9, RSAD2, TLR7
BP	GO:0006633	fatty acid biosynthetic process	0.006735	4	LPL, GGT5, ALOX12, MIF
BP	GO:0046394	carboxylic acid biosynthetic process	0.006891	5	LPL, GGT5, KYNU, ALOX12, MIF
BP	GO:0016053	organic acid biosynthetic process	0.006891	5	LPL, GGT5, KYNU, ALOX12, MIF
BP	GO:0046456	icosanoid biosynthetic process	0.009951	3	GGT5, ALOX12, MIF
BP	GO:0006636	unsaturated fatty acid biosynthetic process	0.011894	3	GGT5, ALOX12, MIF
BP	GO:0008015	blood circulation	0.012860	5	TCAP, OLR1, MYOF, TPM1, CXCL10
BP	GO:0003013	circulatory system process	0.012860	5	TCAP, OLR1, MYOF, TPM1, CXCL10
BP	GO:0051270	regulation of cell motion	0.014554	5	COL18A1, NRP1, THBS1, TPM1, CXCL10
BP	GO:0046903	secretion	0.015412	6	STX1A, LMF1, USE1, CCL5, RAB27A, CXCL10
BP	GO:0016192	vesicle-mediated transport	0.021730	8	STX1A, RABEP2, HIP1R, USE1, TRAPPC5, CCL5, THBS1, RAB27A
BP	GO:0002252	immune effector process	0.027651	4	CARD9, RSAD2, TLR7, RAB27A
BP	GO:0002684	positive regulation of immune system process	0.028807	5	CARD9, IL13RA1, THBS1, TNFRSF4, TLR7
BP	GO:0002544	chronic inflammatory response	0.028923	2	CCL5, THBS1
BP	GO:0045087	innate immune response	0.029812	4	KYNU, TLR7, RAB27A, MIF
BP	GO:0051240	positive regulation of multicellular organismal process	0.031168	5	CARD9, CCL5, THBS1, TPM1, TLR7
BP	GO:0045785	positive regulation of cell adhesion	0.034610	3	THBS1, TPM1, ALOX12
BP	GO:0006968	cellular defense response	0.035673	3	CCR3, CCL5, TNFRSF4
BP	GO:0033273	response to vitamin	0.041171	3	ALPL, KYNU, PMF1
BP	GO:0002694	regulation of leukocyte activation	0.047354	4	IL13RA1, THBS1, TNFRSF4, MIF
BP	GO:0031349	positive regulation of defense response	0.049365	3	CARD9, CCL5, TLR7
BP	GO:0030334	regulation of cell migration	0.049479	4	COL18A1, THBS1, TPM1, CXCL10
BP	GO:0000267	cell fraction	0.002965	13	STX1A, KYNU, CYB5R2, CYP2J2, OLR1, HIP1R, SLC6A4, CCL5, CPT1A, PYGM, HRH4, ABCC4, CA4
CC	GO:0005886	plasma membrane	0.009603	27	ALPL, STEAP4, NRP1, IFITM3, SLC6A4, SLC16A12, RSAD2, TNFRSF4, TPM1, HRH4, TGM3, THBS1, IL13RA1, MYOF, RAB27A, LPL, STX1A, OLR1, ZP3, FADD, GGT5, TARP, CCR3, CA4, ABCC4, TREM2, ALOX12
CC	GO:0044433	cytoplasmic vesicle part	0.010180	5	STX1A, HIP1R, ABCC4, THBS1, RAB27A
CC	GO:0030659	cytoplasmic vesicle membrane	0.025142	4	STX1A, HIP1R, ABCC4, RAB27A
CC	GO:0044421	extracellular region part	0.027958	10	COL18A1, LPL, OGN, ZP3, PLA2G7, PMF1, CCL5, THBS1, MIF, CXCL10
CC	GO:0005624	membrane fraction	0.028767	9	STX1A, CYP2J2, OLR1, HIP1R, HRH4, SLC6A4, ABCC4, CA4, CPT1A
CC	GO:0012506	vesicle membrane	0.031088	4	STX1A, HIP1R, ABCC4, RAB27A
CC	GO:0005615	extracellular space	0.034524	8	COL18A1, LPL, PLA2G7, PMF1, CCL5, THBS1, MIF, CXCL10
CC	GO:0005626	insoluble fraction	0.034688	9	STX1A, CYP2J2, OLR1, HIP1R, HRH4, SLC6A4, ABCC4, CA4, CPT1A
CC	GO:0030141	secretory granule	0.048228	4	STX1A, ABCC4, THBS1, RAB27A
MF	GO:0019955	cytokine binding	0.013930	4	CCR3, IL13RA1, THBS1, TNFRSF4
MF	GO:0008144	drug binding	0.028489	3	PYGM, SLC6A4, TLR7
KEGG PATHWAY	hsa04620	Toll-like receptor signaling pathway	0.029157	4	FADD, CCL5, TLR7, CXCL10

### qRT-PCR validation of DEGs in high and low milk yielders

**Fig. 5:**
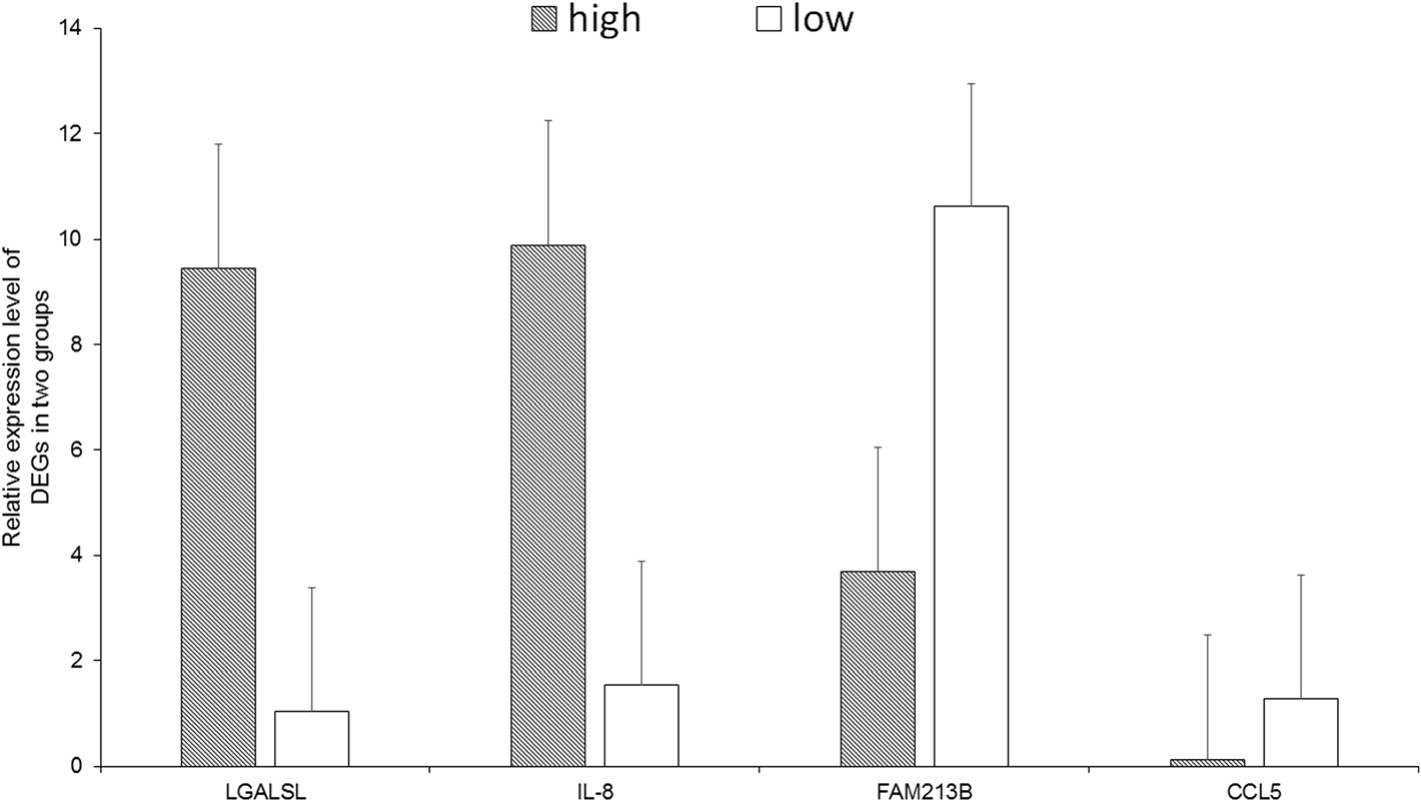
The expression level of DEGs validation by qRT-PCR. *LGALSL* and *IL-8* were highly expressed in high yielders. *FAM213B* and *CCL5* were highly expressed in low yielders detected by RNA-seq

Furthermore, we randomly selected 4 DEGs identified from the RNA-seq data, *LGALSL, IL-8, FAM213B* and *CCL5,* to validate their expression patterns using qRT-PCR. The results from the qRT-PCR confirmed that the DEGs had the same expression pattern observed with the RNA-seq (Fig. 5). This indicates that the gene expression observed in blood transcriptome between high yielders and low yielders was highly credible.

## Discussion

The object of this study is to investigate potential molecular biomarkers in whole blood related to milk production traits in lactating dairy cows, with the aim of putting forward a non-invasive method that identifies the DEGs for milk performance traits. In this study, whole blood genome transcriptome profiles of high and low yield milk cows were investigated using RNA-seq technique. RNA-seq has many advantages over traditional cDNA microarray technologies and it can easily detect low-abundance genes [22, 23]. Marioni et al. demonstrated that RNA-seq and qRT-PCR have a high correlation, and that the Pearson correlation could reach 0.929 [23], which means RNA-Seq is accurate and reproducible.

Among these 100 DEGs, many genes were also detected by other studies associated with milk yield, such as *BOLA-DQA1. BOLA-DQA1* is one of the primary histocompatibility complex (MHC) class II molecules that plays an important role in the immune system. It is thought that *MHC* genes indirectly affect milk production traits by increasing the disease resistance of the cows. In this present study, the expression level of *BOLA-DQA1* was higher in high milk yielders than in low milk yielders. Other research has reported that the *BOLA-DQA1* gene was associated with resistance to mastitis progression [24].

Splicing event analysis showed that DEGs in high and low milk yield cows were different. In low yielders, cows are more inclined to take the ES pattern but in high yielders A3’SS was more likely. Oxidized low-density lipoprotein receptor (*OLR1*), which can degrade the oxidized form of low-density lipoprotein and plays important roles in fatty acid transport, was reported as a potential gene for milk-fat percentage and milk fat yield [25]. The splice site analysis in our study also found that AS in *OLR1* was significant. There were 5 splice sites of *OLR1* in high milk yield including one ES, one A5’SS and three other events. But in low yielders, there was only one splice site on *OLR1, which was A5’SS.* Also, the expression level of *OLR1* was higher in high yielders than in low yielders. Splice events analysis of DEGs can reveal differences between high and low milk yield cows. Among those DEGs containing splice sites, gene ontology enrichment indicated that the PPAR signaling pathway was significantly different between high and low groups. Study showed that *PPARA* is expressed in heart, liver, adipose tissue, and muscle tissue and is involved in fatty acid catabolism [26]; PPARs play important roles in the regulation of metabolic and inflammatory signaling pathways [26, 27]. It is also reported that *PPAR-γ (PPARG)* is over-expressed in adipose tissue and macrophages and primarily regulates adipogenesis [27, 28]. *PPAR-γ* has been reported to significantly increase its expression during lactation in bovine mammary gland [28]. Also, anti-inflammatory properties were observed between *PPARA* and *PPARG* [29]. In our study, we found that this PPAR pathway is also in the blood transcriptome, and it was only detected in high yielders. Moreover, these three PPAR pathway DEGs: *LPL, OLR1,* and *CPT1A,* were highly expressed in high yielders. Also, the Toll-like receptor signaling pathway existed only in high yielders. The Toll-like receptor pathway is also involved in innate immunity [29]. These results indicate that the regulation of metabolic and immune function is more active in high milk yield cows. The immune system plays a key role in health maintenance, pathogenesis, diseases resistance, and production performance. Further research is required to explore the relation between immune response and milk performance traits in cattle blood transcriptomes.

## Conclusion

The present study provides a non-invasive method to identify the DEGs in cattle blood using RNA-seq for milk yield traits. The study revealed 100 DEGs between high yielders and low yielders, and discovered different alternative splicing patterns between the two groups. The enrichment analysis also revealed that specific metabolic and immunological pathways are related to cattle milk yield traits, and could be considered a signature of blood biomarkers selection in dairy cattle. These results provide the valuable resources of biological research in Chinese Holstein cows milk production, but also offer some potential guidelines to understand the relationship between milk production and the immune function.

## Methods

### Blood samples collection

Twenty three Chinese Holstein cows in their second or third lactation were selected based on their current lactation and previous lactation records from BingZhouHai Dairy Farm. All the cows are reared under the same standard. 13 high yielders: ~28kg/day/cow and 10 low yielders: ~18kg/day/cow were selected for this study. 5ml of whole blood was drawn from the jugular vein of each cow. The blood samples were frozen in liquid nitrogen then transferred to −80 °C for further RNA extraction. All experimental methods used in this study were approved by the Inner Mongolia Agricultural University (Hohhot, China) Institutional Animal Care and Use Committee.

### RNA extraction and sequencing

We used TRIzol reagent (TaKaRa, USA) to extract total RNA from blood samples following the manufacturer’s instructions. RNA degradation and purity were monitored on 1% agarose gels. Illumina TruSeq Stranded mRNA Sample Preparation Kit (Illumina, San Diego, CA, USA) was used to generate cDNA libraries according to the manufacturer’s recommendations. A total of 1μg of high quality RNA from each individual was used to prepare the sequencing library. The poly-T oligo-attached magnetic beads selection procedure was used to obtained mRNAs from the total RNA. After fragmentation, random oligonucleotides and SuperScript II was used to synthesize first-strand cDNA. DNA polymerase I and RNase H was used to synthesis Second-strand cDNA. After adenylating of 3’ ends of DNA fragments, Hybridization was initiated by ligating Illumina PE adapter and index. cDNA fragments (200 bp) were generated by the AMPure XP system in purifying the library. DNA fragments were selectively enriched to construct the final sequencing library using Illumina PCR Primer Cocktail [11]. An Illumina Hiseq 2000 platform sued to sequence the libraries.

### Differentially expressed genes and splice events analysis between high and low yielders

Clean reads were acquired by removing low quality reads with adapter and poly-N from raw reads and were used for subsequent analysis. We downloaded the cattle genome (UMD3.1.66) and annotation files from the ensembl database. Bowtie v0.12.8 was used to build the index of cattle reference genome [22] and TopHat v2.1.0 [23] was employed to align clean reads for each sample against the cattle reference genome. DEGs between the high and low yielders were detected using Cuffdiff [30]. P value <0.05 and FDR<0.05 were as the threshold in this study to select differentially expressed genes between high and low yielders.

The Alternative Splicing Transcriptional Landscape Visualization Tool (Astalavista) web server (http://genome.crg.es/astalavista/) extracts and displays splice events from genomic annotation of exon-intron gene coordinates. Astalavista v3.0 [20] was employed to identify the alternative splice events for all available transcripts and to study the five basic splice events of 100 DEGs for 23 samples.

## Enrichment analysis of DEGs

To further investigate the function of 100 DEGs, we performed the gene ontology (GO) and Kyoto Encyclopedia of Genes and Genomes (KEGG) pathway analysis using DAVID bioinformatics resource tool [21]. GO and KEGG terms with p<0.05 were considered significantly enriched.

### Validation of DEGs by qRT-PCR

DEGs identified by the RNA-seq method were validated using qRT-PCR. *GAPDH* was used as the internal quality control. RT-PCR experiments were performed with 2×SYBR Green master mix technology (Takara) on the Mx3000P Real-Time PCR System (Agilent, USA). The reaction was performed using the following program: 95 °C for 10 min; 40 cycles of 95 °C for 15s and 61 °C for 60s; 95 °C for 30s, 55 °C for 30s and 95 °C for 30s. Primer sequences can be found in Additional file 3: Table S3.

## Abbreviations

A3SS, alternative 3’ splicing site; A5SS, alternative 5’ splicing site; AS, Alternative splicing; BP, biological process; CC, cellular component; DEG, differentially expressed gene; ES, exon skipping; EST, expressed sequence tag; FDR, false discovery rate corrected p values; FPKM, Fragments per kilobase of transcript per million mapped fragments; GO, Gene ontology; IR, intron retention; KEGG, Kyoto Encyclopedia of Genes and Genomes; MF, molecular function; MXE, mutually exclusive exon; RNA-Seq, RNA-Sequencing

## Additional files

Additional file 1: Table S1. The total transcripts analyzed in investigated samples. (XLSX 2461 kb) Additional file 2: Table S2. The detail information of 100 DEGs between high and low milk yield cows. (XLSX 19 kb) Additional file 3: Table S3. Primer sequences used for qRT-PCR. (DOCX 14 kb)
