# Supplementary material for: Whole blood transcriptional profiling comparison between different milk yield of Chinese Holstein cows using RNA-seq data
Source: BMC Genomics. 2016 Aug 22;17(Suppl 7):512. doi: 10.1186/s12864-016-2901-1 (PMC5001199; doi:10.1186/s12864-016-2901-1)
Supplement: Additional file 3: Table S3. — Primer sequences used for qRT-PCR. (DOCX 14 kb) [file 12864_2016_2901_MOESM3_ESM.docx]

| **Gene Name** | **Primers Sequence (5'-3')** | **Size (bp)** | **Tm** |
| --- | --- | --- | --- |
| GAPDH | Forward: CACCCTCAAGATTGTCAGCA  Reverse: GGTCATAAGTCCCTCCACGA | 103 | 61°C |
| *LGALSL* | Forward: TCAGAAGATCCTCCTGCCGA  Reverse: TCGCTGACTGTTCTTCACCC | 112 | 61°C |
| IL-8 | Forward: ACTGGCTGTTGCTCTCTTGG  Reverse: GGTGGAAAGGTGTGGAATGT | 125 | 61°C |
| FAM213B | Forward: CTACCTGGACGAGAGCAAGC  Reverse: GCAGCCACTTCACGAACAG | 108 | 61°C |
| *CCL5* | Forward: GAGGACGCCTTGAACCTGAA  Reverse: GTGGAATCTGTGCCTTCCCA | 119 | 61°C |

**Table S3. Primer sequences used for qRT-PCR**

The genes with selection random were identified by qRT-PCR. The housekeeping gene, *GAPDH* was taken for internal control. The data was analyzed by C(t) method.
